# A Pilot Study on the Use of Low Doses of CBD to Control Seizures in Rare and Severe Forms of Drug-Resistant Epilepsy

**DOI:** 10.3390/life12122065

**Published:** 2022-12-09

**Authors:** Gabriela Pesántez Ríos, Luciana Armijos Acurio, Ruth Jimbo Sotomayor, Victor Cueva, Ximena Pesántez Ríos, Hugo Navarrete Zambrano, Samuel Pascual, Galo Pesántez Cuesta

**Affiliations:** 1 Servicio de Neurología Pediátrica, Centro Nacional de Epilepsia, Quito 170121, Ecuador; 2 Departamento de Neurociencias, Universidad Autónoma de Madrid, 28029 Madrid, Spain; 3 Centro de Investigación para la Salud en América Latina (CISeAL), Pontificia Universidad Católica del Ecuador, Quito 170530, Ecuador; 4 Departamento de Radiología, Hospital de Pediatría J.P. Garrahan, Buenos Aires C1245, Argentina; 5 Dirección de Investigación, Pontificia Universidad Católica del Ecuador, Quito 170525, Ecuador; 6 Servicio de Neurología Pediátrica, Hospital Universitario La Paz, 28046 Madrid, Spain

**Keywords:** cannabidiol, CBD, drug resistant epilepsy, seizures

## Abstract

Due to its anticonvulsant properties, cannabidiol can be supportive as an adjuvant therapy in the management of drug resistant epilepsy. This retrospective observational study evaluates the intensity and frequency of the seizures of patients with drug-resistant epilepsy that have been treated with antiepileptic medication associated with CBD in low doses for at least 12 months. Thirty-four patients were included in the study. The most frequent diagnosis of epilepsy was focal symptomatic epilepsy and Lennox–Gastaut syndrome (35.2%). During the follow-up, there was a statistically significant decrease in the seizure frequency (t student *p* < 0.001). A high proportion of patients, 16, concluded the study with a total control of the seizures reaching a 100% improvement, 12 reported ≥ 75% improvement, 3 ≥ 50%, and 2 ≥ 25%; only 1 patient had an improvement of less than 25%. This is the first Latin American study that demonstrates that long-term CBD added to the usual drugs significantly reduces the frequency, duration, and type of seizures in the different etiologies of epilepsy, being especially effective on the seizures that are the most incapacitating, improving the quality of life of the individual and their family.

## 1. Introduction

Refractory epilepsies occur in 30 to 40% of patients with epilepsy. They are characterized by complex clinical histories, frequent and severe seizures, as well as drug resistance. This leads to a greater risk of severe psychomotor delay [1,2,3].

The use of cannabidiol has generated a great interest in the scientific community since it lacks a psychoactive effect [2,4], and as an adjunctive therapy in the treatment of refractory epilepsies, due to its anticonvulsant properties [1,2] and its profile polypharmacological as a neuromodulator, anti-inflammatory, and antioxidant. 

Experimental studies have shown that the endocannabinoid system may be altered in people with epilepsy [2]. Recurrent seizures and the epileptogenic process themselves decrease the expression of the CB1 receptors, and a reduction in the expression of DAGLα (an enzyme responsible for the synthesis of 2-AG) and anandamide [5] levels is also evidenced. 

According to some authors, the administration of cannabidiol could help to regulate the endogenous endocannabinoid system and modulate the level of neuronal excitability. The indirect activation of the endocannabinoid system seeks to decrease the release of excitatory neurotransmitters, and stimulate the synthesis of endocannabinoids themselves or, in turn, inhibit the reuptake of anandamide [6,7]. Additionally, in the short and long term, CBD modifies the epileptogenic activity and the threshold to generate seizures [8,9,10], acting also on other receptors such as GPRR55 and TRPV1 [11,12,13].

There is a substantial amount of scientific evidence around the world of patients who, with the administration of CBD, have achieved control of their seizures. In June 2018, the FDA approved the first pharmaceutical substance derived from cannabis for the treatment of refractory epilepsies [14]. Currently, pure CBD is indicated for the treatment of seizures in Lennox–Gastaut syndrome or Dravet syndrome, in patients older than 2 years, as it is effective, safe, and well tolerated [15,16].

However, in Latin America, the availability of this drug is scarce, and the cost is unaffordable for most of the population. Our objective for this work is to present the experience of the National Center for Epilepsy of Ecuador that treats children, adolescents, and adults diagnosed with drug-resistant epilepsy with CBD for at least 12 months, with the aim of achieving a better control of the seizures and offering an improvement in the quality of life of the patient with epilepsy and their family.

## 2. Materials and Methods

### 2.1. Study Design 

This retrospective observational descriptive study evaluates the intensity and frequency of seizures of patients with drug-resistant epilepsy that have been treated with antiepileptic medication associated with CBD for at least 12 months.

### 2.2. Population

The study includes all the patients treated with antiepileptic medication associated with CBD for at least 12 months at the National Center for Epilepsy (NCE) in Quito, Ecuador. 

The inclusion criteria were: (i) patients of any age group diagnosed with epileptic syndromes catalogued by the International League Against Epilepsy (ILAE) as a drug-resistant epilepsy; (ii) patients that have attended at the National Center for Epilepsy in Quito, Ecuador between 2015 and 2020; and (iii) patients that have been treated with cannabidiol (CBD) for at least 12 months as an adjuvant treatment. 

The exclusion criteria were: (i) patients that have not been diagnosed with drug-resistant epilepsy and (ii) patients that have not been treated with CBD.

### 2.3. Bias

Individuals who have decided not to participate in the study could have had some distinguishing characteristics compared to those who did. To minimize this risk, the basic sociodemographic variables of those who did not wish to participate were collected.

### 2.4. Data Sources 

For this study, we used secondary data collected by the health professionals at the NCE throughout the years of follow-up of their patients. We used hand-written medical records and diagnostic test results as our source of information. 

### 2.5. Medical Records 

The medical records start with a baseline in-depth interview of the patient’s caregivers, followed by all the relevant details related to use of medication and CBD, including seizure types (generalized, focal, convulsive, and non-convulsive) and the frequency of the seizures that the patient or caregiver reports. 

### 2.6. Use of Cannabidiol 

The CBD used by the population in our study was sublingual PhytoCBD Oil at two different doses: (i) 3% CBD oil with 0.02% THC and (ii) 15% CBD oil with 0.02% THC, administered twice a day with an average dose of 1 mg/kg/day, ranging from 0.32 to 2.4 mg/kg/day. 

### 2.7. Statistical Analysis 

Descriptive statistical analysis was carried out in the present study. The statistical software STATA 14.2 was used for the analysis of the data. 

For further analysis, Spearman’s nonparametric test was used to assess possible correlations between the quantitative variables. The value of *p* < 0.05 is calculated as a measure of significance.

We also analyzed the percentage of change in the frequency of seizures in our population. We considered as the ‘number of seizures before CBD use’ as the average number of daily seizures during the last three months before starting the use of CBD. The ‘number of seizures after CBD use’ was considered to be the average of daily seizures presented during the three month period between each evaluation. 

To calculate the average seizures post-CBD, we used the following equation:

seizures post-CBD = (average seizures reported at month 1 + 3 + 6 + 9 + 12)/5(1)

The percentage of change in the seizures was calculated using the following equation:

% of change in seizures = (average seizures before CBD use − average seizures post-CBD) × 100 (2)

To calculate the duration of the seizures, we categorized the variables ‘seizure duration before CBD use’ and ‘seizure duration after CBD use’ into <1 min 1–5 min, and >5 min. Any seizure lasting over 10 min was considered to be ‘Status epilepticus’.

### 2.8. Ethical Concerns 

Following Ecuadorian regulations, observational studies based on secondary data do not need an approval by an IRB when information is properly anonymized, as is our case. 

## 3. Results

There were 34 patients included out of an initial sample of 70 patients. Thirty-six of the patients that were not considered for the study were excluded for different reasons. Three patients abandoned the treatment due to a self-report of an increase in the number of seizures they experienced during the stage of the dose increase, therefore, they were also excluded from the study. The patients were part of the study for only 1–3 months. 

**Figure 1:**
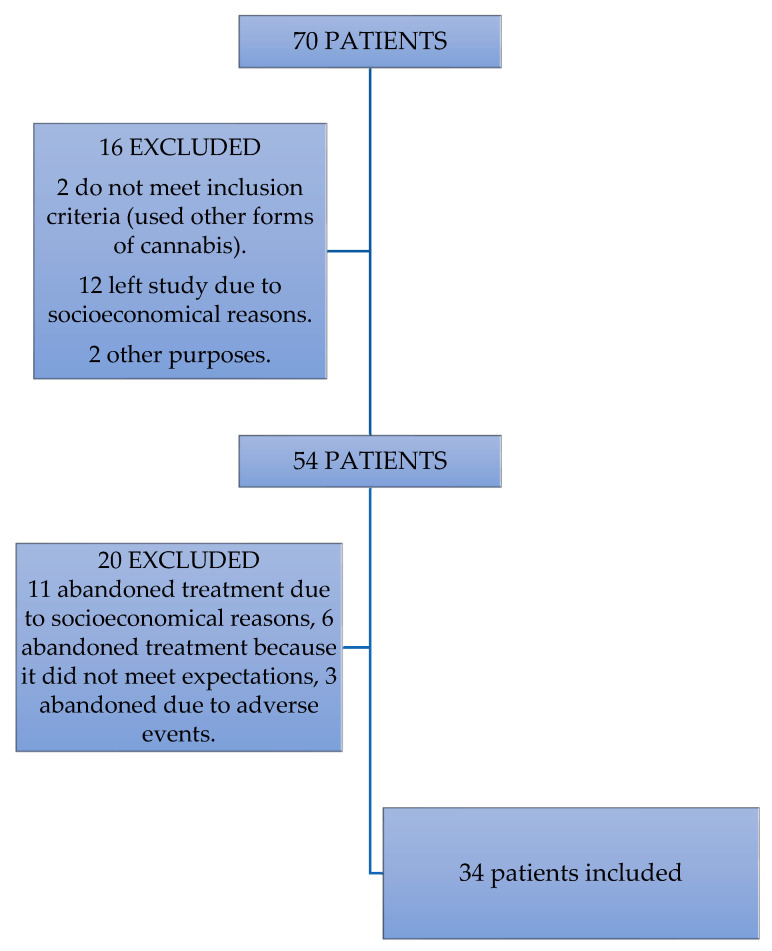
Exclusion of patients.

Six patients decided to stop using cannabidiol as an adjuvant treatment due to family beliefs of what it could cause, disregarding the actual effects of the cannabidiol. We excluded them from the study due to the lack of continuity. Eleven patients were excluded due to either a lack financial support that did not allow them to go the follow-up sessions, leading to a discontinued use of the treatment. The details are presented in Figure 1.

The general parameters of the patients included in the study are presented in Table 1. The most frequent diagnosis of epilepsy is focal symptomatic epilepsy and Lennox–Gastaut syndrome (35.2%). The number of pharmacological treatments received by the patients previous to the use of CBD ranged from 0 to 6, with a mean of 2 treatments.

### 3.1. Frequency of Seizures

The variation in the number of seizures throughout the months of treatment with CBD is presented in Table 2. Prior to the start of CBD, there were 12 (35.29%) patients who presented 1–5 seizures per day, 5 (14.7%) who presented 6–10 seizures per day, 7 (20.6%) with 11–20 seizures per day, 1 (2.9%) presenting 21–30 seizures per day, and 8 (23.5%) presenting > 30 seizures per day. Once the treatment started, a clear reduction was observed in patients presenting 6–10 seizures per day from 5 to 3, from 11–20 seizures per day from 7 to 1, and those with > 30 seizures per day decreased from 8 to 0 patients. At the end of the study, all patients presented less than 10 seizures per day and 16 patients (47.06%) no longer present epileptic seizures.

### 3.2. Duration of Seizures

The duration of the seizures throughout the CBD treatment in patients is plotted below. It can be seen that at the beginning of the study, all patients presented seizures of a varying duration, the most frequent being 1–5 min in 18 (52.94%) patients, followed by <1 min in 13 (38.24%) patients. Furthermore, it was observed that at the beginning of the study, three (8.82%) patients presented seizures greater than 5 min. Subsequently, it can be observed that at the 12-month follow-up, 16 (47.06%) patients without seizures predominate, followed by 12 (35.29%) patients who present seizures of <1 min. At the end of the study, patients with seizures lasting more than 5 min were no longer observed. Results presented in Table 3. 

We analyzed whether there was a difference between the intensity of the seizures in the patients at the beginning of CBD use and at the 12-month follow-up. A statistically significant decrease in the intensity of the seizures was found at the end of the follow-up period (chi2 = 5.44, *p* = 0.0196).

### 3.3. Type of Seizures

**Figure 2:**
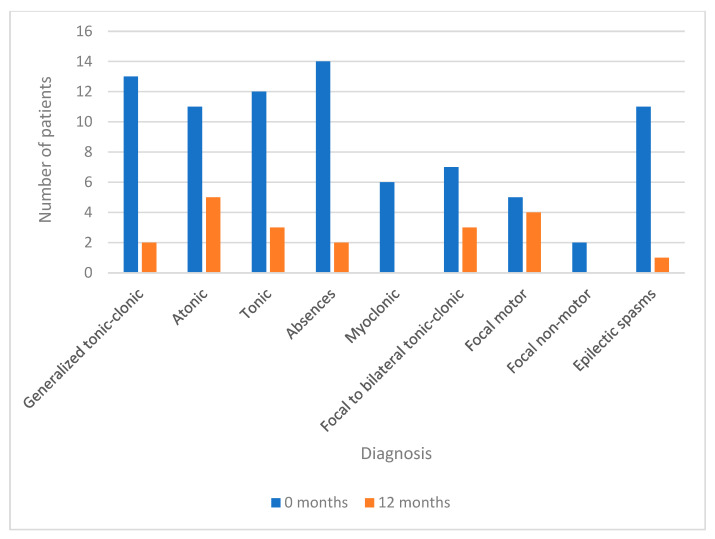
Types of seizures.

At the beginning of the treatment, most patients (N = 25) presented multiple types of seizures, observable in Figure 2, while at the end of the study, the group of patients with single seizures (N = 12) predominated, without seizures (N = 16), and only three patients continued to present associated various types of seizures.

In relation to the type of seizure that manifests itself in each patient before and after the treatment with CBD, it is observed that prior to the treatment, the most common seizures are atypical absences in 14 patients, GCT in 13 patients, tonic in 12, spasms and atonic in 11 patients each.

At the 12-month point, there is a statistically significant difference (*p* < 0.001) between the seizures presented prior to the use of CBD and those presented at the 12-month follow-up. They significantly reduce the motor and generalized seizures, absences in two patients, GCT in two patients, tonic in three patients, atonic in five patients, and spasms in one patient. It is also possible to observe how myoclonic and non-motor focal seizures disappear at the 12-month point in patients who previously presented them.

**Figure 3:**
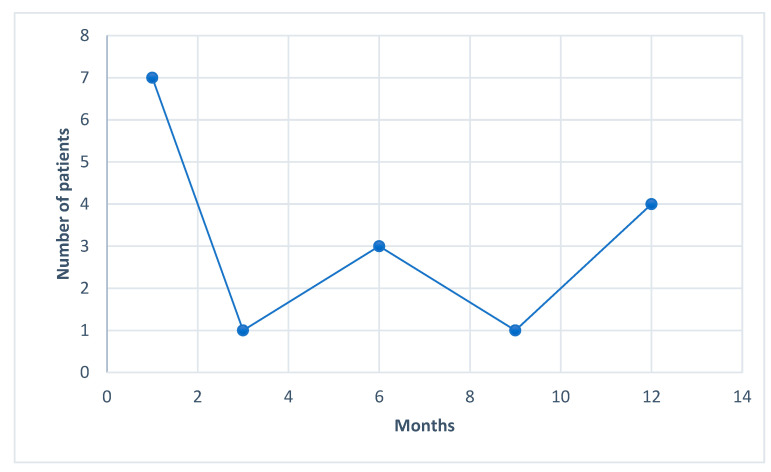
Number of months until patients experienced complete seizure freedom.

We were able to see a tendency towards complete seizure control starting at the first month after the CBD treatment. All the way up to the end of follow-up, we kept observing new patients that reached the goal of zero seizures, as show in Figure 3. 

## 4. Discussion

This a pilot study that includes children and adults with diagnoses of drug-resistant epilepsies who use pure CBD adhered to their basic treatment in low doses (<2.5 mg/kg/day).

There was no statistically significant difference between the sex, age of the patients, type of epilepsy or etiology, and the decrease in the frequency or duration of seizures when using cannabidiol. 

A high proportion of patients, 16, concluded the study with a total control of their seizures, reaching a 100% improvement, 12 reported a ≥ 75% improvement, 3 ≥ 50%, and 2 ≥ 25%; only 1 patient had an improvement of less than 25%. During the follow-up, these changes were evident at each visit and remained consistent over time.

The 15 subjects who still presented seizures at the end of the study also reported a statistically significant improvement (chi2 = 5.44 *p* = 0.0196), since, although a total resolution has not been achieved, the seizures have changed, are less intense, and are shorter in duration. Twelve patients report seizures lasting less than 1 min, and none have seizures lasting more than 5 min.

An important contribution of our study has been to recognize the type of seizures on which CBD is most effective. We confirmed a wide spectrum of action on various types of seizures [17,18], with statistically significant changes (*p* < 0.001) on all types of seizures: generalized tonic-clonic, atonic, tonic, myoclonic, absences, spasms, and focal with a secondary generalization. 

Most of the patients (N = 25) prior to the use of CBD presented multiple types of seizures; during the follow-up, we could see how the seizures of severe and disabling falls disappeared and only those of a short duration and which were mild in intensity or focal seizures remained, predominating the group of 12 patients with single seizures versus the 3 patients who continued to present various types of seizures.

Devinsky and Thiele have described similar results in studies carried out in patients with Lennox–Gastaut syndrome, in whom they show a significant reduction in the frequency of all seizures, mainly on motor and falling seizures [15,19].

The effect of CBD on the presentation of the status epilepticus is not clear. However, we have noticed that the recurrence of these decreases in each period of time which was analyzed. This has even influenced the global perception that patients and caregivers have of an improvement in their epileptological condition, since by reducing the recurrence of status, this has repercussions in less hospital admissions and impacts on a better quality of life for the patient and the patient’s family group [15].

It has been interesting to note that most of these patients prior to the treatment with CBD had tried a range of 0–6 drugs (a mean of 2), in addition to two patients who had undergone callosotomy, treatments for epilepsy with which they had not been able to achieve a significant control of their seizures with, such a control that is demonstrated in this study. It is reported in the literature that, despite the number of available treatments, including non-pharmacological ones such as: ketogenic diet, vagus nerve stimulator, and surgery (resection and callosotomy), less than 10% of patients are seizure-free [15].

Our results in terms of effectiveness are similar to those reported by other research groups and represent an achievement in the treatment of refractory epilepsies [19,20]. In our experience, the use of low doses of CBD (<2.5 mg/kg/day) associated with other AEDs has been well tolerated and the adverse effects presented have been minimal. However, this research contrasts with other CBD studies in which the doses used are greater than 10–20 mg/Kg/day and in which there are a greater number of adverse effects [21,22]. It is likely that the lower incidence of adverse effects in our study is closely related to dose, since an increase in the incidence of drowsiness and diarrhea has been reported, with doses > 10 mg/kg/d, especially at the beginning of the treatment [19,21]. 

Therefore, we corroborate Landmark’s recommendations for a “slow start” and “increase based on individual response” strategy. The milestone of the dosage will depend on the percentage of response in the control of the seizures, so a rigorous observation time of the basal frequency of the seizures is necessary before the administration of CBD [23].

In this way, the dose of CBD remained stable once a clinical improvement was achieved during the 12 months of the follow-up (mean 1 mg/kg/d and a range of 0.32–2.4), which shows that CBD is safe and well tolerated. We have not found a common dose of effectiveness within this group of patients, however, the use of minimal doses of enriched CBD has been possible since its sublingual mode of administration probably avoids the first-pass effect due to the hepatic metabolism and in this way, its bioavailability increases, and it is also clear that pharmacogenetics is essential to be understood in a mainly Hispanic population group. Landmark describes a sub-group of Asian patients with specific genotypic characteristics (polymorphisms in CYP2 C19) that make them poor metabolizers and in whom a low dose of CBD could be effective or sufficient to cause unexpected adverse effects [23].

Therefore, CBD doses continue to be a challenge for new lines of research due to their great interpersonal variability, in addition to other analysis factors such as the effect of the cumulative dose and the time of exposure to the drug [23,24].

In terms of the use of other drugs, it was observed that 58.8% of the patients decreased the number of drugs associated with CBD, this being a determining factor according to some authors in influencing the perception of a better QoL [25].

The decrease in the ACTH requirement during the study period, changing from 18 to 2 patients (Chi2 McnEMAR = 12.8, *p* < 0.001), has also been relevant. This suggests a possible anti-epileptogenic effect of CBD, which in the long term modifies and stabilizes the neural network [15]. In addition to a possible synergistic effect of ACTH and CBD supported by the neurobiological mechanisms of inflammation that underlie epilepsy [26].

Most patients (N = 17) associate an average of three drugs, of which the most frequent combinations with CBD have been CLB, LEV, VPA, and TPM. The minimum effective doses of CBD make its association with other drugs safe, however, it is recommended to analyze the interaction between them since their bioavailability could be affected. Observed in Table 4.

It has been described that CBD increases the effectiveness of other drugs such as CLB and TPM due to the fact that they share the same metabolization cycle, causing the inhibition of CYP2 C19 that favors the increase in their active metabolites, which could exert a synergistic effect that results in a better control of seizures [16,21]. However, it has also been described that due to this interaction, even with adequate doses of the drugs, an increase in their serum levels could be caused, reaching toxic levels with consequent severe adverse effects. This analysis is difficult to clarify as there are currently no studies comparing CBD without using other AEDs.

On the other hand, the interaction of VPA with CBD is studied, due to the possible elevation in liver enzyme levels of being up to more than three times the normal limit. This pharmacokinetic interaction has been seen mainly in patients taking CBD at doses of 20 mg/kg/d, although it has also occurred in rare cases in patients taking CBD without associating VPA. This effect has so far been reversible [21], however it is advisable to monitor transaminases (TGO-TGP), in addition to being attentive to symptoms such as fever, rash, nausea, abdominal pain, and increased bilirubin’s [22,24].

And, the association with Levetiracetam has not reported serum variations of the drug.

Finally, we still do not understand the whole factors involved in the process of pharmacoresistance. However, cannabidiol could be modulating the environment of neuroinflammation and the dysfunction of the hematoencephalic barrier through blockade of the overexpression of ABC transporters which would facilitate the access of AEDs to the central nervous system [27].

## 5. Conclusions

In conclusion, our results show that in the long-term, CBD added to the usual drugs manages to significantly reduce the frequency, duration, and type of seizures in the different etiologies of epilepsy, being especially effective on the seizures that most incapacitate patients, and for this reason, it may improve the quality of life of the individual and their family.

There are some limitations in this study. Furthermore, most of the patients in this study are Hispanic race. There is a possibility that different results will be seen in other races due to differences in drug pharmacokinetics.

The subjects of this study are of a middle social, economic, and cultural level, which has an important impact on the access to and ability to continue with the treatment. Consequently, a significant number of patients who, even knowing and managing to control seizures with CBD, have had to discontinue treatment due to family myths, mobility, and financial difficulties. Taking into account the length of the follow-up, the dropout rate of 24.2% is not surprising, and is within the range of other long-term AED studies, that is 20% to 40% at approximately 1 year of the follow-up [22].

## Figures and Tables

**Table 1:** General characteristics of the patients included in study.

General Description	N = 34
**Sex**	
Female	15 (44.1%)
Male	19 (55.9%)
**Ethnicity**	
Hispanic	33 (97.05%)
Indigenous	1 (2.9%)
**Age**	
Median = 5.3 years	
Range = 0.1–40 years	
**Place of birth**	
Coastal region	3(8.82%)
Mountainous region	30 (88.23%)
Amazon region	1 (2.9%)
**Diagnosis of epilepsy**	
Focal symptomatic epilepsy	12 (35.2%)
Familial myoclonic epilepsy	1 (2.9%)
Continuous partial epilepsy	1 (2.9%)
Possible Dravet syndrome	1 (2.9%)
Doose syndrome	1 (2.9%)
Lennox–Gastaut syndrome	12 (35.2%)
Ohtahara syndrome	1 (2.9%)
West syndrome	5 (14.7%)
**Neuroimaging alterations**	
Yes	30 (88%)
Median = 2 anomalies	
Range = 0–5 anomalies	
No	2 (6%)
No results	2 (6%)
**Number of pharmacological treatments before and after CBD**	
Average before CBD (range)	3 (0–5)
Average after CBD (range)	3 (0–4)
**Householder**	
Work that requires higher education	7 (20.6%)
Work that does not require higher education	22 (64.7%)
Does not work	5 (14.7%)

**Table 2:** Frequency of seizures reported throughout CBD treatment.

Time Consuming CBD (Months)	Frequency of Daily Seizures (N = 34)													
	0		>0–<1		1–5		6–10		11–20		21–30		>30	
**0**	0	(0%)	1	(2.94%)	12	(35.29%)	5	(14.7%)	7	(20.6%)	1	(2.9%)	8	(23.5%)
**1–3**	7	(20.59%)	5	(14.71%)	15	(44.1%)	3	(8.8%)	1	(2.9%)	1	(2.9%)	0	(0%)
**6–9**	14	(41.18%)	5	(14.71%)	11	(32.4%)	3	(8.8%)	1	(2.9%)	0	(0%)	0	(0%)
**12**	16	(47.06%)	4	(11.76%)	7	(20.6%)	4	(11.8%)	0	(0%)	0	(0%)	0	(0%)

**Table 3:** Duration of seizures throughout CBD treatment.

Duration of Seizures	Time Consuming CBD (Months)					
	0		6		12	
**No seizures**	0	(0%)	12	(35.29%)	16	(47.06%)
**<1**	13	(38.24%)	14	(38.24%)	11	(35.29%)
**1–5**	18	(52.94%)	5	(14.71%)	4	(14.71%)
**>5**	3	(8.82%)	1	(2.94%)	0	(0%)

**Table 4:** Combination of drugs used at the end of the study besides CBD.

Combination of Drugs	Number of Patients
NONE	4
LEV/CLB/CBZ	1
LEV/CLB/VPA/TPM	1
LEV/CLB/TPM	1
LEV/CLB/VPA	1
CLB/VPA	1
LEV	4
LEV/CLB	1
LEV/TPM	1
LEV/CLB/VPA	2
LEV/CLB	1
LEV/CLB/GBP	1
LEV/CLB/TPM	1
LEV/TPM	1
LEV/CLB/TPM/LCM	1
LEV/CLB/VPA/TPM	1
LEV/TPM/VPA	1
LEV/CLB/TPM	1
LEV/CLB/TPM	1
VPA/TPM/LTG	1
VPA	2
VPA/CBZ/TPM	1
VPA/CBZ/TPM	1
LEV/CLB/VPA	1
LEV/CLB/VPA/TPM	1
VPA/LTG	1

Drugs: CBZ: Carbamazepine; CLB: Clobazam; GBP: Gabapentin; LCM: Lacosamide; LEV: Levetiracetam; LTG: Lamotrigine; OXC: Oxcarbazepine; TPM: Topiramate; VPA: Valproic acid.

## Data Availability

Not applicable.
